# cano-wgMLST_BacCompare: A Bacterial Genome Analysis Platform for Epidemiological Investigation and Comparative Genomic Analysis

**DOI:** 10.3389/fmicb.2019.01687

**Published:** 2019-07-24

**Authors:** Yen-Yi Liu, Ji-Wei Lin, Chih-Chieh Chen

**Affiliations:** ^1^Central Regional Laboratory, Center for Diagnostics and Vaccine Development, Centers for Disease Control, Taichung, Taiwan; ^2^Institute of Medical Science and Technology, National Sun Yat-sen University, Kaohsiung, Taiwan; ^3^Rapid Screening Research Center for Toxicology and Biomedicine, National Sun Yat-sen University, Kaohsiung, Taiwan

**Keywords:** molecular typing, next generation sequencing, whole-genome multilocus sequence typing, feature selection, epidemiological investigation, comparative genomic analysis

## Abstract

With the decreasing cost of next-generation sequencing, whole-genome sequence-based bacterial genome comparisons are expected to become a mainstream process in the public health domain. Extended multilocus sequence typing (MLST) methods are becoming increasingly popular for use in comparing bacterial genetic relatedness in epidemiological investigations. Several extended MLST schemes based on biological signatures have been reported. Among them, whole-genome MLST (wgMLST) has gradually become one of the most widely used approaches for bacterial strain typing. In addition to using bacterial typing, many researchers aim to identify differences in the genes of compared strains. Because these differences might provide insights into critical bacterial functions, such as virulence and pathogenicity, researchers usually study these genes that differ between strains. Hence, we designed a web service tool based on wgMLST-constructed tree topology coupled with the feature selection method to create the “canonical wgMLST (cano-wgMLST) tree.” The genes that differ between strains are shown at each split of the tree, thereby directly providing information for performing a comparative genomic analysis for each strain pair. We demonstrated that this web service tool could be operated efficiently on two datasets consisting of 22 *Campylobacter jejuni* isolates and 59 *S.* Heidelberg isolates, respectively. We imposed this tool on a designated web server, cano-wgMLST_BacCompare, to enable users to create a wgMLST tree and canonical wgMLST tree automatically from their uploaded bacterial genomes for not only epidemiological investigation but also comparative genomic analysis. Additionally, detailed information on how to use this service is provided. The cano-wgMLST_BacCompare is available at http://baccompare.imst.nsysu.edu.tw.

## Introduction

The origin of the multilocus sequence typing (MLST) approach can be traced back to 1998, when it was proposed by Maiden et al. (1998) as a method for overcoming the typing data exchange problem. By constructing a reference database containing alleles for seven housekeeping gene loci, researchers can easily exchange information regarding isolated strain types by generating a standardized allele profile (sequence type), which consists of a serial number obtained from comparisons made using the reference database. Although *Neisseria meningitidis* was the first species used to test the MLST approach, the approach has been proven to be applicable to many bacterial species, and the information of at least 100 species has been included in MLST allele databases (Jolley and Maiden, 2010; Jolley et al., 2018). After its development, the MLST approach rapidly gained popularity within the public health community. Several studies have demonstrated the successful detection of epidemiological isolates by using the MLST approach (Mellmann et al., 2011; Perez-Losada et al., 2013; Ludeke et al., 2015).

Because schemes selected by the MLST approach contain only few housekeeping-related gene loci, their discriminatory power may not be sufficient for distinguishing closely related isolates. Therefore, various schemes that recruit more gene loci, such as ribosomal MLST (Jolley et al., 2012) and plasmid MLST (Garcia-Fernandez et al., 2008; Carattoli et al., 2014), have been developed. Although MLST-based approaches are useful to public health researchers, the costs of MLST experiments were high in the Sanger sequencing era. With the reduction in cost of next-generation sequencing, many studies have attempted to evaluate whole-genome MLST schemes for comparing the genomes of several common pathogenic bacteria, such as *Salmonella* Enteritidis (Pearce et al., 2018), *Listeria monocytogenes* (Chen et al., 2016), *Vibrio parahaemolyticus* (Gonzalez-Escalona et al., 2017), *Enterococcus faecium* (de Been et al., 2015), and *Campylobacter jejuni* (Cody et al., 2017). These studies have tended to extend the MLST scheme from housekeeping genes to whole-genome genes (i.e., whole-genome MLST and wgMLST) and demonstrated that wgMLST has favorable discriminatory power for distinguishing highly closely related strains. The difficulty in comparing strain types across different laboratories is a limitation of the SNP-based approach used for molecular typing. However, this limitation can be overcome by using the wgMLST approach for bacterial strain typing. Beyond typing, many public health researchers have aimed to identify genes that exhibit differences among compared strains, because these differences may provide insights into critical bacterial functions, such as pathogenicity and virulence. Therefore, a tool that can be used for investigating genomic differences might be helpful for researchers.

In this study, by using the feature selection approach, we developed a user-friendly web server named cano-wgMLST_BacCompare to assist users in building a genetic relatedness tree based on the extracted canonical wgMLST gene set for not only epidemiological investigations but also functional investigations. We designed a two-layered feature selection approach for filtering loci. In the first layer, the whole-genome scheme is extracted for user-uploaded genome sequences. In the second layer, the “feature importance algorithm” is applied for selecting the most critical loci that possess a definite distinguishing ability. The cano-wgMLST_BacCompare web server is a user-friendly platform that can be used for performing comparative analyses. This web server mainly integrates the extraction of the whole genomes and identification of the most discriminatory loci. In addition, the genetic relatedness tree and heatmap profile indicating different genes for each split on the basis of the final created scheme are displayed on the result page.

## Methods and Implementation

**FIGURE 1:**
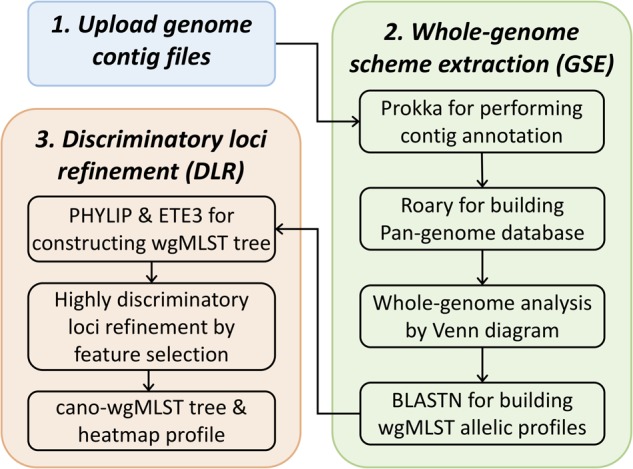
Schematic work flow of cano-wgMLST–BacCompare.

The cano-wgMLST_BacCompare server employs two major processes, namely whole-genome scheme extraction (GSE) and discriminatory loci refinement (DLR), that use the “feature importance” algorithm (Geurts et al., 2006). In the GSE process, a pipeline containing the “contig annotation” and “pan-genome allele database (PGAdb) creation” is used. In the DLR process, “feature importance levels with forests of trees” combined with a binary tree-traversal process were used to identify highly discriminatory loci for each selected split. The workflow for the cano-wgMLST_BacCompare server is displayed in Figure 1, and the details of methodologies used in this server are provided in the following sections.

### GSE

#### Contig Annotation

We utilized a Prokka v1.11 (Seemann, 2014) pipeline to complete the annotation of contigs uploaded by users. The Prokka pipeline is a tool used for rapid prokaryotic genome annotation. The output file format of the annotation process is a “gff” file containing sequences and annotations to be used in the next step.

#### PGAdb Creation

A Roary v3.10.2 pipeline (Page et al., 2015) was used to process a large set of genomes. In this procedure, a “gff” file was used to arrange proteins into orthologous clusters. Paralogous genes were not included in the pan-genome data set. All orthologous clusters included a protein family with a 95% sequence identity. One locus (gene) represented one protein family. The “ffn” format files created in the previous step were used to convert proteins in each cluster into nucleotide sequences for establishing a pan-genome allele data set. In the case that one or more nucleotides failed to provide a suitable match, sequences in a locus were defined as different alleles. The pan-genome allele data set was presented using a matrix table. Loci were encoded using the beginning three letters of genomes followed by a seven-digit number (e.g., SAL0000001or SAL0000002), where integers from one to *n* indicated the number of alleles in each locus.

### DLR

#### wgMLST and cano-wgMLST Profiling

The uploaded genome contigs were compared with the constructed PGAdb by using BLASTN v2.2.30+ (Altschul et al., 1990) program with a minimum identity of 90% and coverage greater than 90% for the locus assignment and a exactly match for the allele assignment. The output indicated whether an allele was present in a locus. If alleles were absent, then “0” was assigned; if an allele was present, then the pre-established allele number was assigned. An “allelic sequence” was constructed after the comparison process.

#### Building a Genetic Relatedness Tree

A genetic relatedness tree was constructed from the previously established allelic sequence by using the clustering algorithm of the unweighted pair-group method with arithmetic averages in the PHYLIP v3.6 program (Felsenstein, 1981). Bootstrap values in a dendrogram were calculated using the ETE3 toolkit (Huerta-Cepas et al., 2016).

#### Feature Selection by “Feature Importance” Algorithm

**FIGURE 2:**
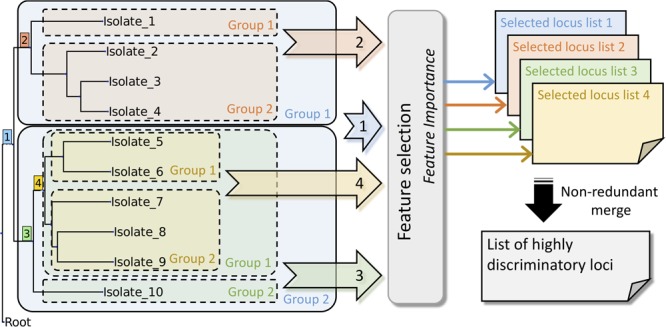
Schematic work flow for identifying highly discriminatory loci.

Feature importance (Geurts et al., 2006) was applied in our study to determine discriminatory loci for each split. To include the features of the highest importance in the data sets, it was necessary to perform artificial classification. To this end, we used the ETE3 toolkit by traversing the Newick format tree produced from the user-selected scheme. The tree-traversal procedure was completed by using our in-house script. After the process, we could acquire a classified data set for performing the feature importance analysis (Figure 2). The feature importance method was based on Extra-Trees algorithms (Geurts et al., 2006), commonly referred to as decision trees (Quinlan, 1986). The classification process used artificial categorizations as targets to be matched using an Extra-Trees module. The feature importance analysis was completed using scikit-learn (Pedregosa et al., 2011), which is a machine learning module in Python. Results included the feature importance rank and magnitude. The schematic work flow of the tree-traversal and feature-extraction procedures are presented in Figure 2.

#### Robinson–Foulds Metric

The Robinson–Foulds (RF) metric (Robinson and Foulds, 1981) is the most widely used measure of phylogenetic tree similarity. Given two phylogenetic trees, the RF metric counts the number of splits or clades induced by one of the trees but not the other. In this study, the RF metric was used to measure the distance between genetic relatedness trees built on the basis of the whole-genome scheme (wgMLST tree) and highly discriminatory loci (cano-wgMLST tree).

### Implementation

The cano-wgMLST_BacCompare server integrates five functional modules: “contig annotation,” “PGAdb creation,” “wgMLST profiling” (containing wgMLST and cano-wgMLST), “genetic relatedness tree construction,” and “highly DLR.” All of these modules were implemented in Perl. The computational task of “feature importance” analysis was completed using scikit-learn (Pedregosa et al., 2011), which is a machine learning module in Python. The webpage was constructed using HTML, JavaScript, PHP, jQuery, and jvenn (Bardou et al., 2014) JavaScript libraries. The server runs on a 24-core Linux cluster with 2.40 GHz Intel Xeon processors.

## Web Server

### Input Format

**FIGURE 3:**
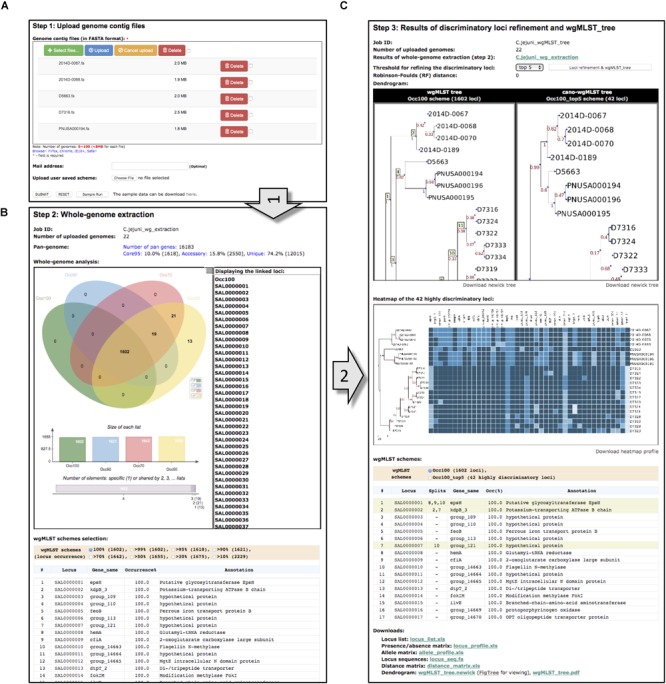
Features of the cano-wgMLST_BacCompare server. **(A)** Input page of the upload genome contig files; **(B)** output page of the whole-genome scheme extraction; **(C)** output page of the highly discriminatory loci refinement and wgMLST tree.

The cano-wgMLST_BacCompare server is initiated by uploading a set of bacterial genome contigs (Figure 3A); then, standard processes, namely contig annotation, PGAdb creation, and wgMLST profiling, are run. The parameter of “protein sequence identity” for PGAdb creation is set at 95%, and parameters for “alignment coverage” and “alignment identity” for wgMLST profiling are both set at 90%. Users are encouraged to provide e-mail addresses to receive notifications when jobs are completed.

### Output Format

The output of GSE comprises the following information. (A) A summary of settings; (B) an illustration of the probability ratio of a locus accounting for core95 genes (occurrence among isolates ≥95%), accessory genes (larger than one allele and <95%), and unique genes (one allele only) in the pan-genome database. The GSE output also includes (C) a four-part Venn diagram with ellipses that illustrates the composition of the whole-genome schemes (Occ100, Occ90, Occ70, and Occ50), marking the inclusion of loci when 100%, 90%, 70%, and 50% of isolates are presented, respectively; (D) a description table for the selected typing scheme; and (E) a button to perform the “loci refinement and wgMLST_tree” function. The output of DLR includes (A) a summary of settings; (B) a wgMLST tree constructed using the user-selected whole-genome scheme; (C) a cano-wgMLST tree constructed using the highly discriminatory loci; (D) Newick tree files to download; (E) a heatmap of the highly discriminatory loci; (F) a description table showing the selected whole-genome scheme and the highly discriminatory loci (labeled in lime); and (G) a summary of output files to download. Examples of GSE and DLR outputs are presented in Figure 3B,C, respectively.

## Examples Analysis

To evaluate the cano-wgMLST_BacCompare, we selected a data set comprising 22 isolates of *C. jejuni* from the benchmark for phylogenetic pipeline validation published by the Centers for Disease Control and Prevention (Timme et al., 2017). For this data set, the *C. jejuni* PGAdb contained 16,183 loci, of which 10.0% (1618 loci) belonged to the core95 genome, 15.8% (2550 loci) belonged to the accessory genome, and 74.2% (12,015 loci) belonged to unique genes (Figure 3B). In this step, we defined the core95 genome as that containing genes present in 95% of the tested genomes, an accessory genome as that containing genes present in two or more but less than 95% of the genomes, and unique genes as those present only in a single genome. The whole genome was further analyzed by constructing a Venn diagram. The PGAdb was then used to construct the wgMLST tree and cano-wgMLST tree and identify highly discriminatory loci for 22 *C. jejuni* isolates by using the DLR module (Figure 3C). In this step, the Occ100 scheme (genes present in all of the tested genomes) was used as the default scheme for constructing the wgMLST tree. The filtered Occ100_top5 scheme (a subset of the Occ100 scheme that unites the five most discriminatory loci for each split) was generated using tree-traversal and feature selection approaches, and the scheme was then used to construct the cano-wgMLST tree. Subsequently, the RF metric was used to measure the distance between the wgMLST tree and cano-wgMLST tree. Finally, the heatmap of highly discriminatory loci was also plotted to easily analyze allele distribution among uploaded isolates. The operation required approximately 1 h for the benchmark data set on a Linux server with 2.40 GHz Intel Xeon processors comprising 24 cores.

**FIGURE 4:**
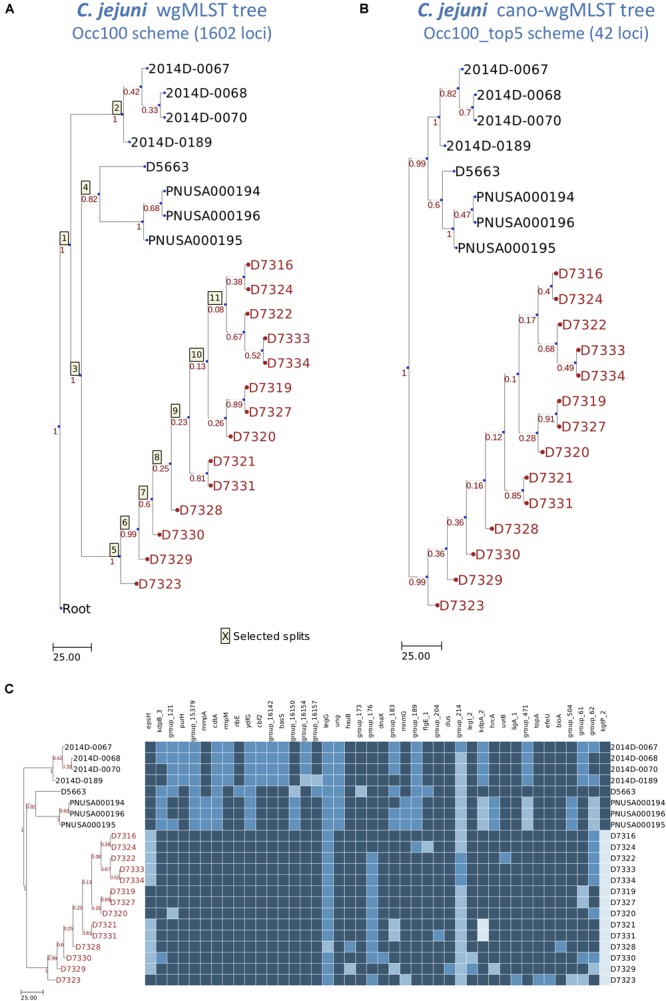
Dendrogram and heatmap constructed using wgMLST profiles for 22 *Campylobacter jejuni* isolates. **(A)** The wgMLST tree generated on the basis of the Occ100 scheme (1602 loci). **(B)** The cano-wgMLST tree generated on the basis of the Occ100_top5 scheme (42 loci). The Occ100 scheme refers to the set of loci that are present in all isolates, and the Occ100_top5 scheme is a subset of the Occ100 scheme that unites the five most discriminatory loci for each split. Outbreak or event-related taxa are colored red. **(C)** The heatmap of 42 highly discriminatory loci. Different alleles in the same column are indicated by different colors.

To analyze 22 *C. jejuni* isolates, 14 outbreak isolates could be clearly grouped, regardless of whether we used the Occ100 scheme (1602 loci) (Figure 4A) or the Occ100_top5 scheme (42 loci) (Figure 4B). The resulting dendrogram of the example data set exhibits high concordance (the measurements of the RF distance is equal to 0) between the wgMLST tree (Figure 4A) and cano-wgMLST tree (Figure 4B). We inferred that the Occ100_top5 scheme (the selected 42 highly discriminatory loci) can sufficiently distinguish between outbreak and sporadic isolates. The selected 42 loci were then used to generate a heatmap profile (Figure 4C). Detailed information of the 42 highly discriminatory loci is provided in Table 1. A heatmap can show how significantly the effect loci are distinguished for each split. By analyzing the heatmap profile, we observed that the genes *cdtA, ydfG, ung*, and *kgtP_2* had potential to be used for distinguishing between outbreak and non-outbreak isolates and that these genes were all selected from split 3 (Table 1). Among them, *cdtA* encoded the cytolethal distending toxin subunit A precursor, which was identified as a virulence-associated gene (Hickey et al., 2000); *ung* encoded uracil-DNA glycosylase, which is likely to be involved in the repair of uracil-containing DNA during base excision repair (Gaasbeek et al., 2009); and *kgtP* encoded α-ketoglutarate permease, which imports a tricarboxylic acid cycle intermediate required for the biosynthesis of glutamate, proline, arginine, and glutamine (Reid et al., 2008).

**Table 1:** List of the 42 highly discriminatory loci selected by 22 *Campylobacter jejuni* isolates.

#	Locus	Splits^a^	Gene name	Annotation
1	SAL0000001	8,9,10	*epsH*	Putative glycosyltransferase EpsH
2	SAL0000002	2,7	*kdpB_3*	Potassium-transporting ATPase B chain
3	SAL0000007	10	*group_121*	Hypothetical protein
4	SAL0000258	1	*purH*	Bifunctional purine biosynthesis protein PurH
5	SAL0000726	3	*group_15379*	Hypothetical protein
6	SAL0000810	4	*mmpA*	Metalloprotease MmpA
7	SAL0000920	3	*cdtA*^b^	Cytolethal distending toxin subunit A precursor
8	SAL0001454	1	*rmpM*	Outer membrane protein class 4 precursor
9	SAL0001455	4	*ribE*	Riboflavin synthase
10	SAL0001471	3	*ydfG*^b^	NADP-dependent 3-hydroxy acid dehydrogenase YdfG
11	SAL0001477	1	*cbf2*	Putative peptidyl-prolyl *cis*-*trans* isomerase Cbf2 precursor
12	SAL0001479	1	*group_16142*	Hypothetical protein
13	SAL0001481	1	*basS*	Sensor protein BasS
14	SAL0001487	4	*group_16150*	Hypothetical protein
15	SAL0001491	2	*group_16154*	Hypothetical protein
16	SAL0001494	2	*group_16157*	Hypothetical protein
17	SAL0001508	8	*legG*	GDP/UDP-N,N’-diacetylbacillosamine 2-epimerase (hydrolyzing)
18	SAL0001515	3	*ung*^b^	Uracil-DNA glycosylase
19	SAL0001524	6,8	*hxuB*	Heme/hemopexin transporter protein HuxB precursor
20	SAL0001525	4	*group_173*	Hypothetical protein
21	SAL0001527	11	*group_176*	Putative type I restriction enzymeP M protein
22	SAL0001529	7	*dnaX*	DNA polymerase III subunit tau
23	SAL0001530	2,9	*group_183*	Hypothetical protein
24	SAL0001531	5	*mnmG*	tRNA uridine 5-carboxymethylaminomethyl modification enzyme MnmG
25	SAL0001534	11	*group_189*	Hypothetical protein
26	SAL0001538	11	*flgE_1*	Flagellar hook protein FlgE
27	SAL0001539	9	*group_204*	Ribonuclease J 1
28	SAL0001540	6	*dus*	Putative tRNA-dihydrouridine synthase
29	SAL0001542	10,11	*group_214*	Hypothetical protein
30	SAL0001543	6,7	*legI_2*	N,N’-diacetyllegionaminic acid synthase
31	SAL0001544	9	*kdpA_2*	Potassium-transporting ATPase A chain
32	SAL0001548	6	*hrcA*	Heat-inducible transcription repressor HrcA
33	SAL0001557	11	*uvrB*	UvrABC system protein B
34	SAL0001560	5	*ligA_1*	DNA ligase
35	SAL0001565	4	*group_471*	Flagellar protein FlaG
36	SAL0001572	5	*topA*	DNA topoisomerase 1
37	SAL0001573	5	*efeU*	Ferrous iron permease EfeU precursor
38	SAL0001578	8	*bioA*	Adenosylmethionine-8-amino-7-oxononanoate aminotransferase
39	SAL0001595	5	*group_504*	Hypothetical protein
40	SAL0001599	7,10	*group_61*	Aminoglycoside 3-N-acetyltransferase
41	SAL0001600	2,6,7,8,9,10	*group_62*	Hypothetical protein
42	SAL0001601	3	*kgtP_2*^b^	Alpha-ketoglutarate permease

*^*a*^The split number can be referenced to the split marker that is labeled in the wgMLST tree (Figure 4A). ^*b*^Selected genes that have the potential to be used for distinguishing between outbreak and non-outbreak isolates.*

Another real case dataset consisting of next generation sequencing data for 59 *S.* Heidelberg, sequenced by Bekal et al. (2016), was also used to evaluate our approach. As illustrated in Supplementary Figure S1, the genetic relationships among the 59 isolates constructed using the cano-wgMLST approach were highly concordant with the relationships of the isolates determined using the high-quality core genome single-nucleotide variant (hqSNV) approach (three foodborne disease outbreaks), as shown in a Bekal’s study (Bekal et al., 2016). The selected 125 loci were then used to generate a heatmap profile (Supplementary Figure S2). Detailed information of the 125 highly discriminatory loci is provided in Supplementary Table S1.

In summary, these examples demonstrate that our cano-wgMLST_BacCompare server not only correctly constructs genetic relatedness trees but also has the potential to select highly discriminatory loci from user-uploaded genome sequences.

## Discussion and Conclusion

The proposed online tool, cano-wgMLST_BacCompare, which comprises two modules (i.e., GSE and DLR), was established to help users conduct epidemiological investigations and comparative genomic analyses involving bacterial whole-genome sequences. A strong advantage of the cano-wgMLST_BacCompare server is its incorporation of the feature selection method to filter the most important loci, which indicate the key genes contributing to the diversity between compared strains for each split along the wgMLST genetic relatedness tree. Because a tree can only be correctly interpreted from an evolutionary viewpoint, many horizontal gene transfer events (e.g., conjugation of plasmids) that might interfere with the correctness of a tree are usually excluded from calculations for the dendrogram. Therefore, it is reasonable to use conserved genetic markers, such as core genes, to construct the skeleton of the genetic relatedness tree; the further locus-reducing process is then applied on the basis of this tree topology. However, since many researchers may need to investigate differences in genes between strains for examining pathogenicity and antimicrobial resistance. Therefore, although our default tree building is based on the core scheme, we also provide different scheme options comprising gene loci with lower occurrences for users to choose from if their aim is mainly to investigate targets usually belonging to accessory genes. We believe that the cano-wgMLST_BacCompare can serve as a powerful online tool for not only epidemiological analysis but also comparative genomic analysis.

## Data Availability

Publicly available datasets were analyzed in this study. This data can be found here: https://github.com/WGS-standards-and-analysis/datasets.

## Author Contributions

Y-YL and C-CC conceived and designed the experiments. All authors performed the experiments, analyzed the data, built analysis tools and webpages, and wrote the manuscript.

## Conflict of Interest Statement

The authors declare that the research was conducted in the absence of any commercial or financial relationships that could be construed as a potential conflict of interest.
