# Supplementary material for: cano-wgMLST_BacCompare: A Bacterial Genome Analysis Platform for Epidemiological Investigation and Comparative Genomic Analysis
Source: Front Microbiol. 2019 Jul 24;10:1687. doi: 10.3389/fmicb.2019.01687 (PMC6668299; doi:10.3389/fmicb.2019.01687)
Supplement: Supplementary file 1 [file Data_Sheet_1.PDF]

cano-wgMLST_BacCompare: A Bacterial Genome Analysis Platform for Epidemiological Investigation and Comparative Genomic Analysis

Yen-Yi Liu [1†], Ji-Wei Lin [2†], and Chih-Chieh Chen [2,3*]

[1] *Central Regional Laboratory, Center for Diagnostics and Vaccine Development, Centers for Disease Control, Taichung 40855, Taiwan*

[2] *Institute of Medical Science and Technology, National Sun Yat-sen University, Kaohsiung 80424, Taiwan*

[3] *Rapid Screening Research Center for Toxicology and Biomedicine, National Sun Yat-sen University, Kaohsiung 80424, Taiwan*

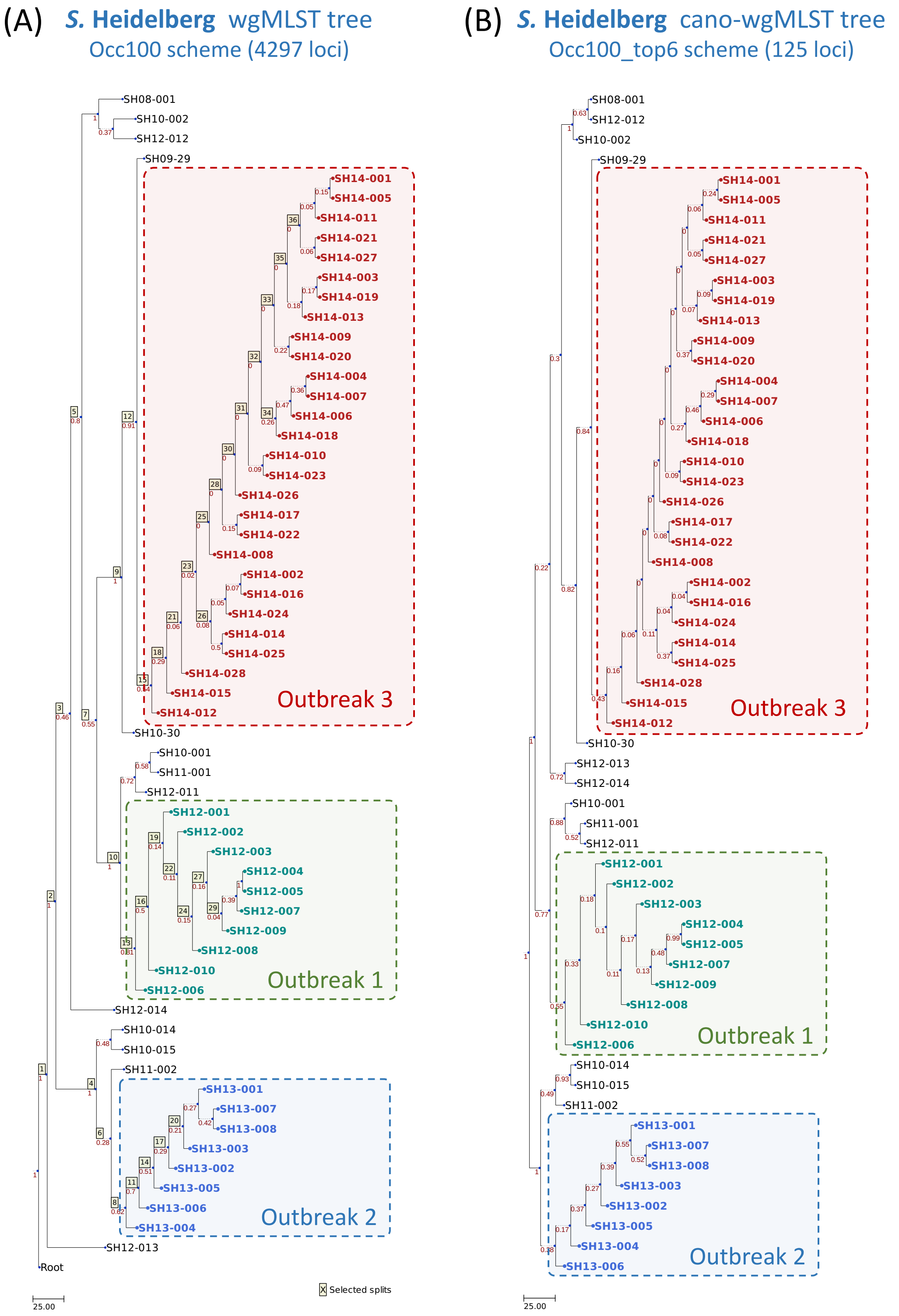

Figure S1. Dendrogram and heatmap constructed using wgMLST profiles for 59 *S.* Heidelberg isolates. (A) The wgMLST tree generated on the basis of the Occ100 scheme (4297 loci). (B) The cano-wgMLST tree generated on the basis of the Occ100_top6 scheme (125 loci). The Occ100 scheme refers to the set of loci that are present in all isolates, and the Occ100_top6 scheme is a subset of the Occ100 scheme that unites the six most discriminatory loci for each split. Isolates for three foodborne disease outbreaks are marked.

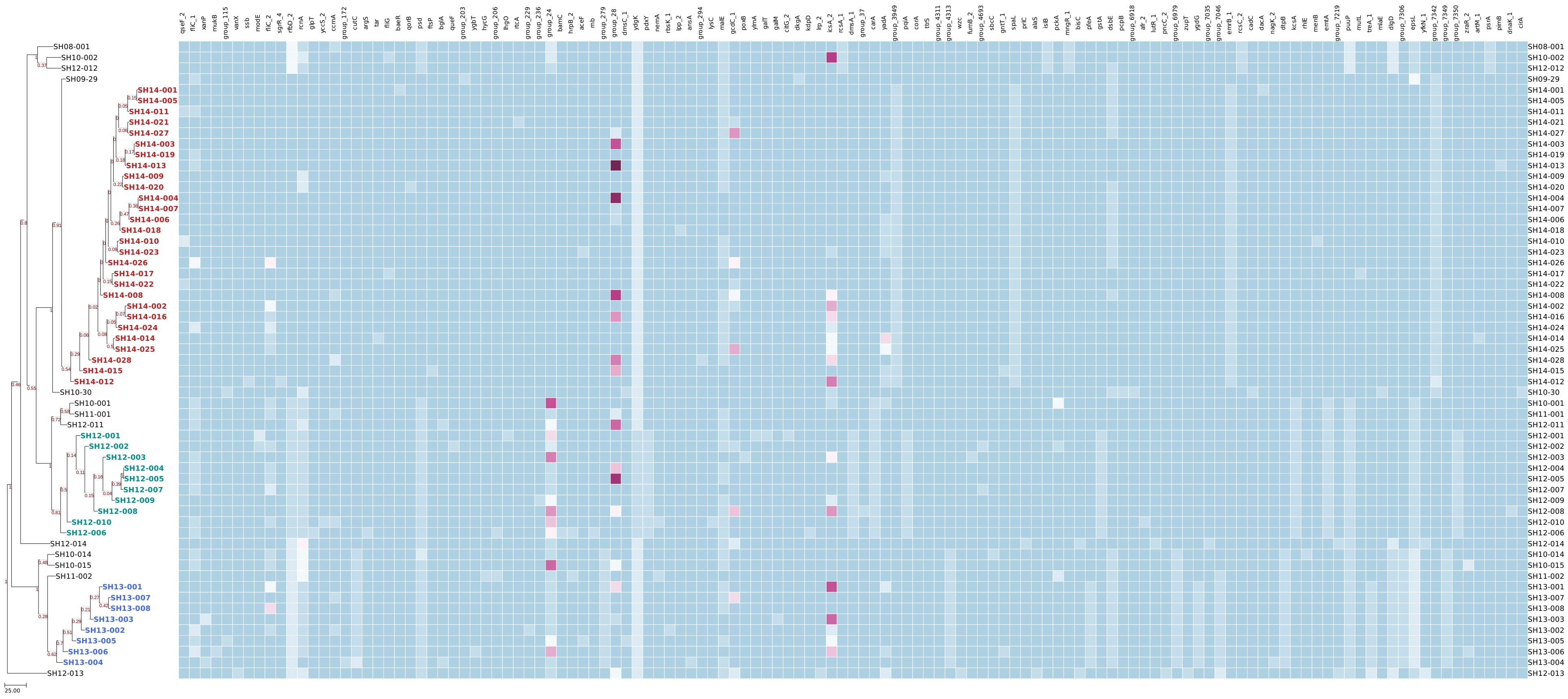

Figure S2. The heatmap of 125 highly discriminatory loci. Different alleles in the same column are indicated by different colors.

Table S1. List of the 125 highly discriminatory loci selected by 59 *S.* Heidelberg isolates.

#	Locus	Splits[a]	Gene name	Annotation
1	SAL0000002	28,30,31,35,36	qseF_2	Transcriptional regulatory protein QseF
2	SAL0000003	14,17,22,23,24,29,30,32,33,35	fliC_1	Flagellin
3	SAL0000004	20	xanP	Xanthine permease XanP
4	SAL0000005	11	mukB	Chromosome partition protein MukB
5	SAL0000006	9,14	group_115	Anaerobic dimethyl sulfoxide reductase chain C
6	SAL0000007	1	vanX	hypothetical protein
7	SAL0000008	15	ssb	Single-stranded DNA-binding protein
8	SAL0000010	19,22	modE	Transcriptional regulator ModE
9	SAL0000011	20,21,23,26,27,29,30	fliC_2	Flagellin
10	SAL0000012	15	sgrR_4	HTH-type transcriptional regulator SgrR
11	SAL0000015	7	rfbD_2	UDP-galactopyranose mutase precursor
12	SAL0000020	8,33	rcnA	Nickel/cobalt efflux system RcnA
13	SAL0000023	13	glpT	Glycerol-3-phosphate transporter
14	SAL0000025	16	yccS_2	Inner membrane protein YccS
15	SAL0000027	16,17,21,25	ccmA	Cytochrome c biogenesis ATP-binding export protein CcmA
16	SAL0000031	8	group_172	putative NUDIX hydrolase
17	SAL0000034	8	cutC	Copper homeostasis protein CutC
18	SAL0000035	13	argS	Arginine--tRNA ligase
19	SAL0000036	26	tar	Methyl-accepting chemotaxis protein II
20	SAL0000040	28	fliG	Flagellar motor switch protein FliG
21	SAL0000044	36	baeR	Transcriptional regulatory protein BaeR
22	SAL0000047	33	qorB	Quinone oxidoreductase 2
23	SAL0000050	7	psd	Phosphatidylserine decarboxylase proenzyme
24	SAL0000057	18	ftsP	Cell division protein FtsP precursor
25	SAL0000058	8	bglA	6-phospho-beta-glucosidase BglA
26	SAL0000060	22	queF	NADPH-dependent 7-cyano-7-deazaguanine reductase
27	SAL0000061	12	group_203	hypothetical protein
28	SAL0000062	11	ygbT	CRISPR-associated endonuclease Cas1
29	SAL0000063	6	hycG	Formate hydrogenlyase subunit 7
30	SAL0000064	6	group_206	Putative multidrug export ATP-binding/permease protein
31	SAL0000065	19	lhgO	L-2-hydroxyglutarate oxidase LhgO
32	SAL0000071	36	rtcA	RNA 3'-terminal phosphate cyclase
33	SAL0000072	17	group_229	hypothetical protein
34	SAL0000079	29	group_236	Acetyltransferase Pat
35	SAL0000083	11,14,16,19,24,27,29	group_24	Y_Y_Y domain protein
36	SAL0000084	13	bamC	Outer membrane protein assembly factor BamC precursor
37	SAL0000099	6,13	hrpB_2	ATP-dependent RNA helicase HrpB
38	SAL0000101	14,31	aceF	Dihydrolipoyllysine-residue acetyltransferase component of pyruvate dehydrogenase complex
39	SAL0000107	13	rnb	Exoribonuclease 2
40	SAL0000121	2	group_279	hypothetical protein
41	SAL0000122	18,20,21,25,29,30,32,33,34,35,36	group_28	hypothetical protein
42	SAL0000124	14	dmsC_1	Anaerobic dimethyl sulfoxide reductase chain C
43	SAL0000125	10	ydgK	Inner membrane protein YdgK
44	SAL0000127	10	pdxY	Pyridoxamine kinase
45	SAL0000128	6,16	nemA	N-ethylmaleimide reductase
46	SAL0000131	17	rbsK_1	Ribokinase
47	SAL0000132	32,34	lpp_2	Major outer membrane lipoprotein Lpp precursor
48	SAL0000134	8	ansA	L-asparaginase 1
49	SAL0000136	21	group_294	Phage P2 GpU
50	SAL0000137	16	lysC	Lysine-sensitive aspartokinase 3
51	SAL0000138	15,17,18,20,25,27,28,31,32	malE	Maltose-binding periplasmic protein precursor
52	SAL0000139	22,23,24,25,26,28,30,35,36	gcdC_1	Glutaconyl-CoA decarboxylase subunit gamma
53	SAL0000140	27	poxB	Pyruvate dehydrogenase [ubiquinone]
54	SAL0000160	19	ylmA	putative ABC transporter ATP-binding protein YlmA
55	SAL0000171	19	galT	Galactose-1-phosphate uridylyltransferase
56	SAL0000182	20	galM	Aldose 1-epimerase
57	SAL0000193	19	citG_2	2-(5''-triphosphoribosyl)-3'-dephosphocoenzyme-A synthase
58	SAL0000221	12	dkgA	2,5-diketo-D-gluconic acid reductase A
59	SAL0000269	13	kdpD	Sensor protein KdpD
60	SAL0000313	1	lrp_2	Leucine-responsive regulatory protein
61	SAL0000412	14,15,17,20,21,23,24,25,26,27,29,31	icsA_2	Outer membrane protein IcsA autotransporter precursor
62	SAL0000676	5	rcsA_1	Transcriptional regulatory protein RcsA
63	SAL0000808	10	dmsA_1	Dimethyl sulfoxide reductase DmsA precursor

64	SAL0000822	24	group_37	hypothetical protein
65	SAL0000943	7	carA	Carbamoyl-phosphate synthase small chain
66	SAL0001023	11,15,18,23,26,31,32,33,34	yadA	Adhesin YadA precursor
67	SAL0001073	12	group_3949	putative kinase inhibitor
68	SAL0001192	10	pqiA	Paraquat-inducible protein A
69	SAL0001429	34	corA	Magnesium transport protein CorA
70	SAL0001430	34	ttrS	Tetrathionate sensor histidine kinase TtrS
71	SAL0001435	34	group_4311	hypothetical protein
72	SAL0001437	2	group_4313	hypothetical protein
73	SAL0001550	1	wzc	Tyrosine-protein kinase wzc
74	SAL0001721	27	fumB_2	Fumarate hydratase class I, anaerobic
75	SAL0001815	22	group_4693	putative assembly protein
76	SAL0002162	4	sbcC	Nuclease SbcCD subunit C
77	SAL0002722	11,18	gntT_1	High-affinity gluconate transporter
78	SAL0002893	12	spaL	putative ATP synthase SpaL
79	SAL0002972	3	prlC	Oligopeptidase A
80	SAL0003002	1	alaS	Alanine--tRNA ligase
81	SAL0003102	5	isiB	Flavodoxin
82	SAL0003520	22	pckA	Phosphoenolpyruvate carboxykinase [ATP]
83	SAL0003667	5	mngR_1	Mannosyl-D-glycerate transport/metabolism system repressor MngR
84	SAL0003995	3	bisC	Biotin sulfoxide reductase
85	SAL0004002	4,6	pfeA	Ferric enterobactin receptor precursor
86	SAL0004005	10	gstA	Glutathione S-transferase GstA
87	SAL0004007	18,21,23,25,28,30,32,33,35	dsbE	Thiol:disulfide interchange protein DsbE
88	SAL0004012	9	pcpB	Pentachlorophenol 4-monooxygenase
89	SAL0004020	9	group_6918	Trypsin
90	SAL0004027	16	afr_2	1,5-anhydro-D-fructose reductase
91	SAL0004028	3	lutR_1	HTH-type transcriptional regulator LutR
92	SAL0004029	1	pncC_2	Nicotinamide-nucleotide amidohydrolase PncC
93	SAL0004061	2	group_6979	Peptidase M16 inactive domain protein
94	SAL0004068	1	zupT	Zinc transporter ZupT
95	SAL0004092	4,6	ygdG	Flap endonuclease Xni
96	SAL0004093	3	group_7035	Prophage CP4-57 integrase
97	SAL0004099	4	group_7046	Intracellular multiplication and human macrophage-killing
98	SAL0004106	12	emrB_1	Multidrug export protein EmrB
99	SAL0004113	5	rcsC_2	Sensor histidine kinase RcsC
100	SAL0004124	9	cadC	Transcriptional activator CadC
101	SAL0004154	36	dacA	D-alanyl-D-alanine carboxypeptidase DacA precursor
102	SAL0004163	8	nagK_2	N-acetyl-D-glucosamine kinase
103	SAL0004169	2	dtpB	Dipeptide and tripeptide permease B
104	SAL0004177	7	kcsA	pH-gated potassium channel KcsA
105	SAL0004178	4	rhlE	ATP-dependent RNA helicase RhlE
106	SAL0004190	31	menB	1,4-Dihydroxy-2-naphthoyl-CoA synthase
107	SAL0004199	7	emtA	Endo-type membrane-bound lytic murein transglycosylase A precursor
108	SAL0004202	3	group_7219	Serine dehydratase alpha chain
109	SAL0004212	7	puuP	Putrescine importer PuuP
110	SAL0004219	28	mutL	DNA mismatch repair protein MutL
111	SAL0004235	4	treA_1	Periplasmic trehalase precursor
112	SAL0004250	9	mlaE	putative phospholipid ABC transporter permease protein MlaE
113	SAL0004252	5	dlgD	2,3-diketo-L-gulonate reductase
114	SAL0004257	2	group_7306	Polysaccharide biosynthesis/export protein
115	SAL0004270	12	epsL	putative sugar transferase EpsL
116	SAL0004279	3	yfkN_1	Trifunctional nucleotide phosphoesterase protein YfkN precursor
117	SAL0004281	15	group_7342	glucose-6-phosphate isomerase
118	SAL0004288	2	group_7349	MbtH-like protein
119	SAL0004289	10	group_7350	hypothetical protein
120	SAL0004290	11	zraR_2	Transcriptional regulatory protein ZraR
121	SAL0004291	26	artM_1	Arginine transport ATP-binding protein ArtM
122	SAL0004292	5	psrA	Polysulfide reductase chain A precursor
123	SAL0004293	35	pimB	GDP-mannose-dependent alpha-(1-6)-phosphatidylinositol monomannoside mannosyltransferase
124	SAL0004294	24	dnaK_1	Chaperone protein DnaK
125	SAL0004296	9	cirA	Colicin I receptor precursor

[a] The split number can be referenced to the split marker that is labeled in the wgMLST tree (**Figure S1A**).
